# A83 PLANT-BASED ENTERAL NUTRITION: A POTENTIAL METHOD OF SECRETION MANAGEMENT IN A PALLIATIVE PATIENT WITH PROGRESSIVE MYOTONIC DYSTROPHY — A CASE REPORT

**DOI:** 10.1093/jcag/gwae059.083

**Published:** 2025-02-10

**Authors:** E F Nap Hill, J Broening, S Sasson, G Ou

**Affiliations:** The University of British Columbia, Vancouver, BC, Canada; St Paul’s Hospital, Vancouver, BC, Canada; The University of British Columbia, Vancouver, BC, Canada; St Paul’s Hospital, Vancouver, BC, Canada

## Abstract

**Background:**

Degenerative neuromuscular conditions such as myotonic dystrophy is characterized by oropharyngeal dysfunction leading to reduced ability to eat orally and clear respiratory secretions. Enteral nutrition (EN) is often required in these patients but aspiration pneumonia remains a concern. Dairy-based EN are commonly used, but evidence suggests that casein digestion may stimulate mucin production in respiratory tissues, increasing secretions (Bartley 2010).

McClanahan et al. (2019) showed that switching patients to plant-based EN improved gastrointestinal outcomes and reduced medication use in chronically ill, tube-fed children. Viall et al. (1990) found soy-based EN led to fewer gastrointestinal issues, such as vomiting and gastric residuals, compared to casein-based EN. Goelen et al. (2021) also reported that plant-based EN resulted in faster gastric emptying compared to casein-based EN, which could reduce secretion-related complications.

**Aims:**

To describe nutrition management in a palliative patient with myotonic dystrophy with recurrent aspiration pneumonia.

**Methods:**

Case report.

**Results:**

A patient with myotonic dystrophy type 1, was admitted with viral respiratory infection resulting in respiratory failure and prolonged hospitalization with marked deterioration in respiratory and swallowing functions. A nasogastric and then percutaneous gastrostomy feeding tubes were placed but feeding regimens remained suboptimal (50% caloric provision) due to recurrent aspiration pneumonia. A transgastric jejunal feeding tube was then placed without much clinical improvement despite trying various enteral feeding formula (including high-protein and high-calorie formulations) and maximal anti-secretory management as per palliative care team. Aggressive feeding was in line with patient’s goals of care after much discussion, so nutrition team was consulted for recommendations.

Patient was switched to plant-based EN due to concerns that dairy protein stimulated secretions. Patient experienced a reduction in secretion thickness and fewer aspiration events. Goal EN rate was achieved.

**Conclusions:**

This case highlights the potential benefits of plant-based EN in managing secretions and reducing aspiration risk in a patient with progressive myotonic dystrophy. Transition from dairy-based to a plant-based alternative led to better secretion control, fewer aspirations, and reduced antibiotics. While this is a single case, the findings align with evidence that plant-based EN may improve gastrointestinal outcomes and alleviate secretion-related complications. Further studies are needed to explore the efficacy of plant-based EN in broader patient populations and its role in managing secretions and aspiration pneumonia.

**Funding Agencies:**

**None**

